# Supplementary figures and images for: Trends in malaria prevalence among school-age children in Mainland Tanzania, 2015–2023: A multilevel survey analysis
Source: PLOS Glob Public Health. 2025 Apr 9;5(4):e0004386. doi: 10.1371/journal.pgph.0004386 (PMC11981166; doi:10.1371/journal.pgph.0004386)

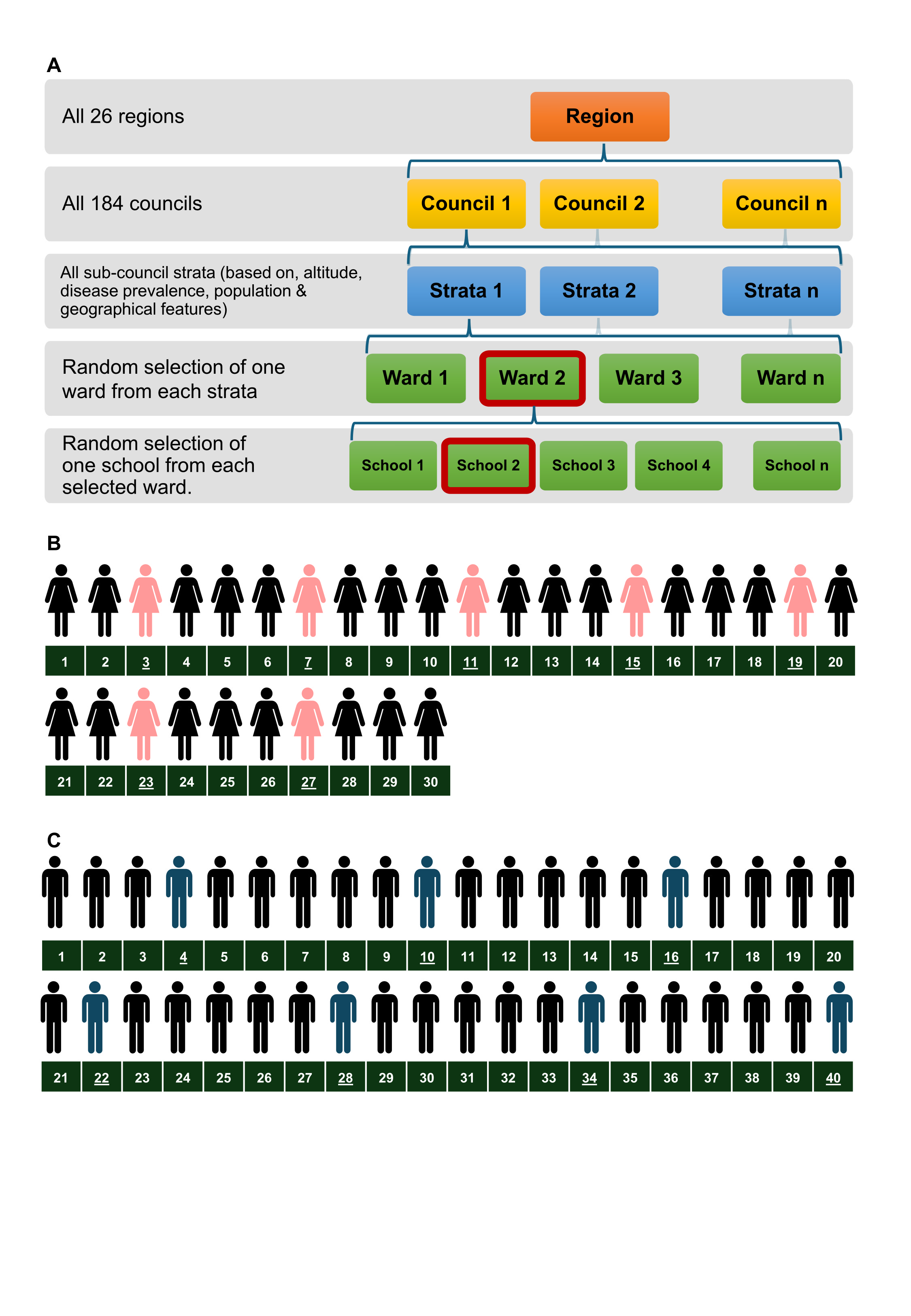

Supplement: S1 Fig — Multistage sampling procedures for Wards, Schools and School Children. The selection of wards, schools, and school children followed a multistage, stratified, proportional probability-to-size sampling procedure. Fig. A illustrates the selection of wards and schools, Fig. B depicts the selection of female children, and Fig. C shows the selection of male children. These figures were created using PowerPoint, and the icons representing male and female figures are licensed under the Creative Commons CC0 1.0 Universal Public Domain Dedication. Links to these icons are as follows: File: Woman - The Noun Project.svg - Wikimedia Commons, File: Man - The Noun Project.svg - Wikimedia Commons. (TIF) [file pgph.0004386.s001.tif]
