# Supplementary material for: Trends in malaria prevalence among school-age children in Mainland Tanzania, 2015–2023: A multilevel survey analysis
Source: PLOS Glob Public Health. 2025 Apr 9;5(4):e0004386. doi: 10.1371/journal.pgph.0004386 (PMC11981166; doi:10.1371/journal.pgph.0004386)
Supplement: S1 Table — Tanzania SMPS data collection period. (DOCX) [file pgph.0004386.s002.docx]

| **Survey round** | **Year** | **Start date (DDMMYY)** | **End date (DDMMYY)** | **Schoolchildren surveyed** |
| --- | --- | --- | --- | --- |
| 1 | 2014 (Phase I) | 11/8/2014 | 18/9/2014 | 11436 |
| 1 | 2015 (Phase II) | 18/5/2015 | 1/6/2015 | 18743 |
| 1 | 2015 (Phase III) | 7/10/2015 | 16/10/2015 | 18934 |
| 2 | 2017* | 24/7/2017 | 13/11/2017 | 64621 |
| 3 | 2019 | 19/8/2019 | 1/9/2020 | 63806 |
| 4 | 2021 | 2/10/2021 | 24/11/2021 | 64959 |
| 5 | 2023 | 2/8/2023 | 14/10/2023 | 65500 |

S1_Table.docx

**The observed long-time data collection in 2017 survey round was due to a release of managerial directives while study teams were already in the field. SMPS team had no control in the situation*
