# Supplementary material for: Trends in malaria prevalence among school-age children in Mainland Tanzania, 2015–2023: A multilevel survey analysis
Source: PLOS Glob Public Health. 2025 Apr 9;5(4):e0004386. doi: 10.1371/journal.pgph.0004386 (PMC11981166; doi:10.1371/journal.pgph.0004386)
Supplement: S2 Table — Trend malaria prevalence with different background characteristics, 2015-2023. (DOCX) [file pgph.0004386.s003.docx]

S2_Table: Trend malaria prevalence

|  | Survey year | | | | |
| --- | --- | --- | --- | --- | --- |
| Characteristic | 2015 | 2017 | 2019 | 2021 | 2023 |
| Malaria RDT positive | N = 10,627 | N = 10,196 | N = 9,151 | N = 7,604 | N = 7,375 |
|  | (95%CI) | (95%CI) | (95%CI) | (95%CI) | (95%CI) |
| Malaria prevalence | 21.6 (21.3 – 22.0) | 15.9 (15.6 – 16.1) | 14.3 (14.1 – 14.6) | 11.8 (11.5 – 12.0) | 11.3 (11.0 – 11.5) |
| Age group (years) |  |  |  |  |  |
| 5 – 8 | 18.4 (17.6 – 19.2) | 12 (11.4 – 12.6) | 11.4 (10.9 – 11.9) | 8.6 (8.1 – 9.1) | 8.6 (8.2 – 9.1) |
| 9 – 12 | 21.9 (21.4 – 22.4) | 15.2 (14.8 – 15.6) | 14 (13.6 – 14.3) | 11.3 (11.0 – 11.7) | 11.4(11.1 – 11.8) |
| 13 – 16 | 23.3 (22.6 – 24.1) | 19.2 (18.6 – 19.8) | 17.6 (17.1 – 18.2) | 15(14.5 – 15.6) | 14.6 (14.0 – 15.2) |
| Sex |  |  |  |  |  |
| Female | 20 (19.5 – 20.5) | 14.4 (14.0 – 14.8) | 13 (12.7 – 13.4) | 10.7 (10.3 – 11.0) | 10.1(9.8-10.5) |
| Male | 23.3 (22.7 – 23.8) | 17.4 (17.0 – 17.8) | 15.7 (15.3 – 16.1) | 12.9 (12.5 – 13.3) | 12.4 (12.0 – 12.7) |
| Slept under ITN last night |  |  |  |  |  |
| No | 28.4 (27.1 – 29.8) | 17.8 (16.9 – 18.6) | 18.4 (16.5 – 20.3) | 16.4 (14.9 – 18.0) | 17 (16.1 – 18.0) |
| Yes | 21.6 (21.2 – 22.1) | 15.4 (15.1 – 15.8) | 13.1 (12.8 – 13.4) | 12.1 (11.6 – 12.6) | 11.1 (10.8 – 11.4) |
| History of Fever |  |  |  |  |  |
| No | 19 (18.6 – 19.4) | 14.3 (13.9 – 14.6) | 21.6(20.3 – 22.9) | 10.6 (10.2 – 11.1) | 10.4 (10.2 – 10.7) |
| Yes | 27.3 (26.6-28) | 19.9 (19.3 – 20.5) | 21.9 (21.0 – 22.8) | 17.7 (16.5 – 19.0) | 18.1 (17.2 – 19.0) |
| Southern | 33.6 (32.2 – 35.1) | 35.4(33.6 – 37.2) | 29.6 (27.9 – 31.4) | 22.4 (21.1 – 23.8) | 16.6 (15.4 – 17.8) |
| Western | 29.3 (28 – 30.6) | 25.7(24.6 – 26.8) | 26.5 (25.5 – 27.6) | 19.7 (18.7 – 20.7) | 16.9 (16.0 – 17.9) |
| Eastern | 17.7 (16.8 – 18.6) | 10.2(9.7 – 10.8) | 8.0 (7.5 – 8.5) | 7.6 (7.1 – 8.1) | 7.9 (7.4 – 8.4) |
| Southwest Highlands | 17.2 (16.1 – 18.2) | 9.1(8.4 – 9.8) | 10.3 (9.5 – 11.0) | 9.9 (9.2 – 10.6) | 8.1 (7.4 – 8.8) |
| Southern Highlands | 12.0 (11 – 13) | 12.5(11.6 – 13.5) | 7.3 (6.5 – 8.1) | 4.4 (3.9 – 5.0) | 6.4 (5.8 – 7.1) |
| Northern | 5.3 (4.7 – 5.8) | 7.3(6.7 – 7.8) | 6.3 (5.8 – 6.8) | 4.1 (3.7 – 4.6) | 3.6 (3.3 – 4.0) |
| Central | 3.0 (2.6 – 3.5) | 1.8(1.5 – 2.1) | 1.9 (1.6 – 2.2) | 0.6 (0.4 – 0.8) | 1.7 (1.4 – 2.0) |
| Elevation group |  |  |  |  |  |
| 0 – <750m asl | 23 (22.3 – 23.8) | 16.9 (16.4 – 17.4) | 14 (13.5 – 14.5) | 12.3 (11.8 – 12.8) | 11.4 (11 – 11.9) |
| 751 – <1250m asl | 27 (26.3 – 27.6) | 20.1 (19.6 – 20.6) | 19.9 (19.4 – 20.4) | 15.9 (15.4 – 16.4) | 14.9 (14.4 – 15.3) |
| 1251 – 1750m asl | 16.6 (16.0 – 17.2) | 12.4 (11.9 – 12.9) | 10.4 (9.9 – 10.8) | 8 (7.7 – 8.4) | 8.4 (8.0 – 8.8) |
| >1,750m asl | 4 (3.3 – 4.9) | 0.9 (0.6 – 1.3) | 1.1 (0.8 – 1.6) | 0.4 (0.2 – 0.7) | 1.0 (0.7 – 1.3) |
