# Supplementary material for: Trends in malaria prevalence among school-age children in Mainland Tanzania, 2015–2023: A multilevel survey analysis
Source: PLOS Glob Public Health. 2025 Apr 9;5(4):e0004386. doi: 10.1371/journal.pgph.0004386 (PMC11981166; doi:10.1371/journal.pgph.0004386)
Supplement: Attachment — Submitted filename: review_PGPH-D-24-01380.pdf [file pgph.0004386.s005.pdf]

Review of Manuscript No PGPH-D-24-01380

Title: Trend malaria prevalence and associated risk factors among school children in mainland Tanzania, 2015-2023: A multilevel analysis of school malaria and parasite surveys

Article Type: Research Article

Keywords: malaria; school age children; Tanzania; malaria transmission; mosquito net use; malaria prevalence; Surveillance; trend analysis

1 General Comments

The article is addressing an important area of public health. Results from surveys of the type conducted here are informative for it gives a clear picture on the health status of the population of a country and at the same time evaluates the effects of control measure usually applied by governments. In the case of malaria, many countries in sub-Saharan Africa do have National Malaria Control Programmes (NMCPs) and it will be very useful to conduct surveillance of the type reported in this article. It is important to recognise that the work presented in the manuscript is cost, and labour intensive, and the output are of far reaching importance.

The present paper reports results of a survey conducted every other year from 2015-2023 among school-aged children 5-16 years in mainland Tanzania. The authors set as objective "... to determine malaria infection prevalence and associated risk factors among school children in mainland Tanzania from 2015 to 2023." The study area and materials and methods are clearly outline and stated. Though I did not fully appreciate the handling of associated risk factors. The language in the manuscript is clear and void of typos. The literature review is rich and

the results of the survey are very important for public health practitioners.

The manuscript, being as it is, a report from a survey; the School Malaria Parasitological Survey (SMPS), in Tanzania, we can only examine the report for consistency. To this effect, I make the following observations and technical comments below, which I believe will strengthen the paper if well addressed.

2 Technical considerations

1. **The Title:** The current title is "Trend malaria prevalence and associated risk factors among school children in mainland Tanzania, 2015-2023: A multilevel analysis of school malaria and parasite surveys".

   (a) I have highlighted those parts of the title that need attention for clarity. What is trend malaria? do you mean "trends in malaria..", What kind of malaria is school malaria? what is school parasite? Maybe we can have something like "A multilevel analysis of surveys for malaria infection in schools.

   (b) The word parasite in the title: Lines 207-211 details the kits (rapid diagnostic kits) used. RDTs are antigen based test and there is no mention of laboratory work to determine actual parasite load (see lines 413-425 where limitations of RDT is stated). So it may be necessary to delimit the use of the word parasite in the title, as it appears to be misleading.

   (c) The aspects of risk factors in the title: I did not see the trends in risk factors in the study. Lines 212-228 presents independent variables that are used to explain the dependent variable. In this section, one would have

expected to see the variables that explain the associated risk factors (we see only age, use of ITN, fever, altitude). It is not clear to me how the trends in associated risk factors was handled.

The title should be adjusted to reflect the work reported in the manuscript. Such an adjusted title could read like: "*Trends in malaria prevalence among school children in mainland Tanzania, 2015-2023: A multilevel survey and analysis* ".

2. Page 4 The abstract: Line 49 we read, "School children play an important role in malaria transmission." It is not clear what the authors want the readers to get from this statement, as it is not evident to me how being a school child becomes important for malaria transmission.

3. Line 33: **Key words**: Key words are too many and overlapping. Maintaining a few such as; *malaria prevalence; Tanzanian Malaria Survey; trend analysis,* may be better.

4. There are repetitions: See, for example, Lines 215-218 and lines 231-233, Line 270 and line 282.

5. Line 274. The logistic regression formula is stated on page 13 and nothing else is said about this formula except that the analysis was done using the R software. Please say something about how this formula was implemented. What were the $X_{k,ij}$ terms in your work? how many terms? etc.

6. Page 14, line 284. Demographic characteristics.... The period of survey was 8 years. Did you examine different kids at all times or some kids participated in the survey more than once. In this case it will be interesting to know if there were repeated cases of malaria infection and among which demographic group of kids. The write-up is silent about

the sampling used. This needs to be clarified. A 5 years old kid in a school at the beginning of the survey would be 13 years at the end and still be legible to enroll in the survey.

7. Lines 364-369: Could the dress code of the boys, in relation to the girls, have been a factor?

8. Line 385: ...ITN usage... not IT usage

9. Lines 461-463: was there a follow-up (tests) to ensure that a child that was positive for malaria actually got treated of their infection, after being given artemether-lumefantrine?

10. **More serious**: The analysis reported and the results seemed very good until one reached the data analysis section of the paper. Errors appear on the tables.

    (a) Table 1. One would expect the data entries for the age groups to add up to the total reported for that year. For example, for the year 2015 we have age-range(number surveyed)= 5-8(9,242), 9-12(26,469), 13-16(13,232). Here $9,242) + 26,469 + 13,232 = 48,943 \neq 49,113$, the total number reported for the 2015 survey. The same kinds of errors show up in looking closely at the data for the other survey years. The same comment applies to the male-female numbers, they do not add up for 2017 and 2021.

    (b) The percentages calculated add up to a hundred meaning that there are issues with the data entries.

    when I started seeing these discrepancies, I stopped checking the other tables. The authors should thoroughly check the data presented and the results reported.

11. Figure 1, where is Tanzania on the map of Africa?

3 Final comments

It is my opinion that this paper is an important survey report and that the results are very useful for calibrating malaria control programmes. It can also be very useful source of parameters to mathematical modellers who write models for malaria transmission. The fact that the prevalence started plateauing from 2021-2023 may be alarming as it is not certain whether the decreasing trend is changing to an increase one. **I will recommend the paper for publication**, but first the issues pointed above should be clarified and specifically the data analysis cleaned up.
