# Supplementary material for: Trends in malaria prevalence among school-age children in Mainland Tanzania, 2015–2023: A multilevel survey analysis
Source: PLOS Glob Public Health. 2025 Apr 9;5(4):e0004386. doi: 10.1371/journal.pgph.0004386 (PMC11981166; doi:10.1371/journal.pgph.0004386)
Supplement: Attachment — Submitted filename: Response to Reviewers.docx [file pgph.0004386.s007.docx]

**Reviewer Comments and Responses**

**1. Title Revision**

•**Reviewer Comment**: The current title needs clarification, particularly phrases like “Trend malaria,” “school malaria,” and “school parasite.” Suggested changes for accuracy.

•**Response**: Thank you for the suggestion. We have revised the title for clarity and accuracy. The new title is:

**“Trends in Malaria Prevalence Among School-Age Children in Mainland Tanzania, 2015-2023: A Multilevel Survey Analysis.”**

**2. Clarification in Abstract on Malaria Transmission Role (Line 49)**

•**Reviewer Comment**: The statement “School children play an important role in malaria transmission” needs clarification.

•**Response**: We agree that clarification is necessary. We revised the sentence to reflect that school-aged children contribute significantly to malaria transmission due to higher prevalence rates in high-transmission settings.

•**Revised Sentence**:

“In high-transmission areas, school-aged children have higher malaria prevalence and contribute significantly to the transmission reservoir.”

**3. Keywords Revision (Line 33)**

•**Reviewer Comment**: Reduce and refine the keywords to remove redundancy.

•**Response**: We have streamlined the keywords to focus on core topics. The revised keywords are malaria, school age children, Tanzania malaria survey, malaria prevalence, trend analysis.

**4. Repetitions in Text (Lines 215-218, 231-233, and 270-282)**

•**Reviewer Comment**: There are repetitive sections that need streamlining.

•**Response**: Thank you for identifying these areas. We reviewed the sections and removed redundancies to improve clarity and flow.

•**Changes**: Consolidated and revised the text regarding the multilevel model setup and the description of predictors in the logistic regression model as follows: “We conducted a multilevel mixed-effects logistic regression analysis to assess malaria infection risk factors, accounting for clustering by school with random intercepts for each school location. This model included both individual-level (age group, sex, history of fever, and mosquito net usage) and school-level predictors (elevation, geographic zone, survey year) as independent variables. Analyses and visualizations were performed in R statistical software version 4.2.3.”

**5. Logistic Regression Model Details (Line 274)**

•	**Reviewer Comment**: Include additional details on implementing the logistic regression formula and specify predictor variables.

•	**Response**: Thank you for highlighting this area. We acknowledge the unclear information of specifying predictor variables used in the formula while implementing logistic regression.

•	**Changes:** We have reviewed the formula as follow: $logit\left( p \right)=\left( \frac{p}{1-p} \right)=\beta_{0}+\beta_{1} . Age_{group}+ \beta_{2} . sex+ \beta_{3} . Elavation_{group}+ \beta_{4} . Zone+ \beta_{5} . \mathrm{History}_{\mathrm{fever}}+ \beta_{6} . \mathrm{Sleeping}_{\mathrm{ITN}}+ \beta_{7} . Survey_{year}+ \mu_{i}$.

Additional interpretation of the formula has been incorporated in the main document.

**6. Sampling and Demographic Clarification (Page 14, Line 284)**

•**Reviewer Comment**: Clarify whether the same children were sampled across survey years and whether repeated cases of malaria infection were tracked.

•**Response**: Although there is a gap of one year between data collection periods, it is possible that some children may have been selected more than once. The Tanzania SMPS sampling strategy was designed to establish a school-based surveillance system, where the lowest unit of analysis is the school rather than individual pupils. However, a separate analysis will be conducted to track the children who appeared in multiple survey rounds, linking their malaria infection status, demographic information, household characteristics, and any changes in malaria-related practices over time.

•**Changes**: Added a clarification in **Line number 150-151, page 8**, “The sampling strategy was designed to establish a school-based surveillance system in the country, where the lowest unit of analysis is the school rather than individual children”.

Also, added a clarification in the discussion section regarding the limitations of repeated sampling and the potential impact on results.

**7. Influence of Dress Code on Malaria Risk (Lines 364-369)**

•**Reviewer Comment**: Consider whether dress code differences between boys and girls could influence malaria risk.

•**Response**: This is an insightful suggestion. Although we did not collect data on dress code, we acknowledged could be a potential confounder and will discuss with stakeholders to include it in future surveys.

**8. Typo Correction on ITN Usage (Line 385)**

•**Reviewer Comment**: Correct the typo from “IT usage” to “ITN usage.”

•**Response**: We have corrected this typographical error.

•**Changes**: Replaced “IT usage” with “ITN usage” on Line 385.

**9. Follow-Up on Malaria Treatment Post-Diagnosis (Lines 461-463)**

•**Reviewer Comment**: Inquire if there was follow-up on malaria treatment adherence post-diagnosis.

•**Response**: We acknowledge that children who tested positive for malaria were provided with antimalarial medicines as recommended by the Tanzania national guidelines for diagnosis and treatment. However, as you clearly noted, there was no mechanism in place for follow-up. This will be taken into account in the next survey rounds, where we will request the school administrations to follow up with the children who tested positive for malaria and were provided with antimalarial medicines.

**10. Correct Data Discrepancies in Tables (Table 1)**

•**Reviewer Comment**: Errors in Table 1 regarding age group and gender totals that do not align with survey totals.

•**Response**: Thank you for bringing this to our attention. After reviewing Table 1, we found that these discrepancies were likely due to missing values. We corrected the totals and recalculated the percentages. We also added a footnote to Table 1 to indicate any missing values.

•**Changes**: Updated Table 1 to correct discrepancies, ensuring all totals align with survey data, and added a footnote to account for missing values.

**11. Tanzania Map Label in Figure 1**

•	**Reviewer Comment**: The location of Tanzania on the Africa map in Figure 1 needs to be clear.

•	**Response**: We updated Figure 1 to clearly label Tanzania within the Africa map.

•	**Changes**: Revised Figure 1 to include a clear label for location of Tanzania on the Africa map.

We appreciate the reviewer’s time and effort in helping us enhance our manuscript, and we hope that these revisions adequately address all comments. We look forward to your further feedback.

Sincerely,

Frank Chacky;

Corresponding and lead Author

National Malaria Control Programme, Ministry of Health, Dodoma, Tanzania

Global Health Institute – Antwerp University, Antwerp, Belgium

Email: frank.chacky@student.uantwerpen.be and frank.chacky@gmail.com

Mobile: +255-75-462-5131
